# Experimental Investigations on the Conductance of Lipid Membranes under Differential Hydrostatic Pressure

**DOI:** 10.3390/membranes12050479

**Published:** 2022-04-29

**Authors:** Rose Whiting, Pangaea W. Finn, Andrew Bogard, Fulton McKinney, Dallin Pankratz, Aviana R. Smith, Elen A. Gardner, Daniel Fologea

**Affiliations:** 1 Department of Physics, Boise State University, Boise, ID 83725, USA; roseywhiting@boisestate.edu (R.W.); pangaeafinn@u.boisestate.edu (P.W.F.); andybogard@u.boisestate.edu (A.B.); fultonmckinney@u.boisestate.edu (F.M.); dallinpankratz@u.boisestate.edu (D.P.); avianasmith@u.boisestate.edu (A.R.S.); elengardner@u.boisestate.edu (E.A.G.); 2 Biomolecular Sciences Graduate Program, Boise State University, Boise, ID 83725, USA

**Keywords:** bilayer lipid membrane, conductance, curvature, pressure, electrophysiology

## Abstract

The unassisted transport of inorganic ions through lipid membranes has become increasingly relevant to an expansive range of biological phenomena. Recent simulations indicate a strong influence of a lipid membrane’s curvature on its permeability, which may be part of the overall cell sensitivity to mechanical stimulation. However, most ionic permeability experiments employ a flat, uncurved lipid membrane, which disregards the physiological relevance of curvature on such investigations. To fill this gap in our knowledge, we adapted a traditional experimental system consisting of a planar lipid membrane, which we exposed to a controlled, differential hydrostatic pressure. Our electrophysiology experiments indicate a strong correlation between the changes in membrane geometry elicited by the application of pressure, as inferred from capacitance measurements, and the resulting conductance. Our experiments also confirmed the well-established influence of cholesterol addition to lipid membranes in adjusting their mechanical properties and overall permeability. Therefore, the proposed experimental system may prove useful for a better understanding of the intricate connections between membrane mechanics and adjustments of cellular functionalities upon mechanical stimulation, as well as for confirmation of predictions made by simulations and theoretical modeling.

## 1. Introduction

Mechanical stimulation from the physical environment is one of the most primeval mechanisms of cellular response [1,2]. The large variety of mechanical stimuli to which cells and complex organisms are continually exposed led to an evolution-directed development of distinct mechano-transduction mechanisms, responsible for processes ranging from sensing changes in osmotic pressure [2,3] to more advanced biological functions, such as touching and hearing [4,5,6]. Irrespective of the specific nature of the mechanical stimulus, the plasma membrane plays a crucial role in mechanical transduction [7]. At the membrane level, mechano-transduction may be achieved by employing specialized receptors and transporters [2,3,7,8,9,10,11]. For example, mechano-sensitive channels [1,3,8] are activated by mechanical stimuli acting upon membranes and mediate the transport of otherwise non-permeant solutes across them. Such behavior is typical for mechano-regulated, catalyzed transport of ions through any cell membrane via ion channels. However, investigations on unassisted ion permeation across lipid membranes are gaining momentum. For a long time, it was considered that physiologically relevant inorganic ions must overcome the very large Born energy barrier presented by the lipid membrane [12,13,14], hence, leading to a very weak permeability. Nonetheless, numerous simulations and experimental investigations indicate inorganic ion permeation through bare lipid membranes [15,16,17,18], which may be easily quantified from conductance measurements carried out in typical electrophysiology experiments on planar bilayer lipid membranes (BLMs) [17]. However, such membranes are flat, and they do not replicate the physical features of curved membranes, which are more relevant from a physiological standpoint. In this respect, simulations performed on an asymmetric and highly curved model of a plasma membrane predict an increase of up to three orders of magnitude for the permeability of water, ions, and anti-cancer drugs [18]. The influence of curvature on a membrane’s structural parameters, such as thickness, order parameter, area per lipid, and cholesterol’s inclination and distribution [19], as well as other physical parameters, were intensively scrutinized by simulations [19,20,21]. Recently, an experimental system consisting of Giant Unilamellar Vesicles with curvature controlled by osmotic pressure was utilized to investigate the direct entry of cell-penetrating peptides [22]. All these theoretical and experimental explorations indicate that a better understanding regarding the influence of mechanical stress on the transport through lipid membranes provides novel cues for deciphering how ubiquitous environmental factors modulate cellular physiology in health and disease. In this line, our work addresses this gap in knowledge by employing an experimental system that facilitates investigations on the transport of inorganic ions through artificial BLMs exposed to a differential pressure, simply achieved by adjusting the height of a liquid column in one of the reservoirs bathing a vertical membrane. Basic electrophysiology measurements of the membrane’s conductance and capacitance reveal that pressure reversibly modulates the membrane’s permeability to monovalent inorganic ions. Further, our experiments confirm the numerous prior observations that the addition of cholesterol to lipid membranes modulates their mechanical and transport properties.

## 2. Materials and Methods

### 2.1. Chemicals

We used two different lipid mixtures to produce starter planar lipid membranes of compositions close to those of mammalian cell membranes. The cholesterol-free membrane was composed of Asolectin (Aso, Sigma-Aldrich, St. Louis, MO, USA) and Sphingomyelin (SM, Avanti Polar Lipids, Alabaster, AL, USA) dissolved in n-decane (TCI America, Portland, OR, USA) as stock solutions (100 mg/mL Aso, and 50 mg/mL SM) and mixed to provide an Aso:SM ratio of 10:4 (*w*:*w*). Cholesterol (Chol, Sigma-Aldrich) was dissolved in n-decane (50 mg/mL) and was used to produce a mixture of Aso:SM:Chol at w:w ratios of 10:4:4. Throughout our experiments we used a buffered electrolyte solution consisting of 135 mM KCl (ThermoFisher Scientific, Waltham, MA, USA) and 20 mM HEPES (pH = 7.2, ThermoFisher Scientific).

### 2.2. Production and Characterization of Planar and Curved Bilayer Lipid Membranes (BLM)

**Figure 1:**
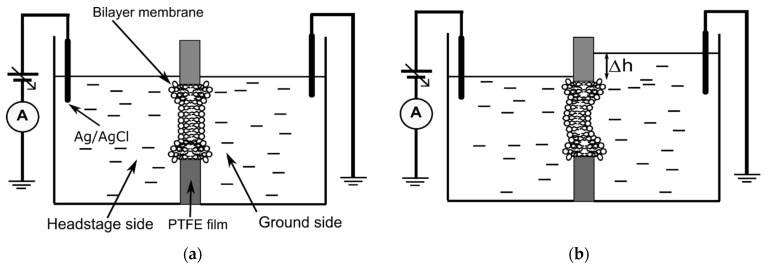
The experimental setup for investigations on ionic transport through lipid membranes exposed to controlled differential pressure. Left (**a**): a planar bilayer lipid membrane is formed in a traditional setup. Right (**b**): the planar membrane is subjected to the pressure created in one reservoir through addition of electrolyte solution. The physical and transport properties of the membranes are monitored by electrophysiology measurements (capacitance and conductance). For both schematics, the Plateau-Gibbs border (annulus) is observed at the lipid membrane-PTFE film interface. The diagrams are not to scale.

The starter planar BLMs were made in a small PTFE film in which we produced a small hole (~100–150 μm diameter) using an electric spark. The film was sandwiched between two reservoirs custom machined from black Delrin^®^ (DuPont, Wilmington, DE, USA), each one capable of holding a total volume larger than 2 mL (see the schematics in Figure 1a). Each reservoir was filled with 0.8 mL electrolyte solution, and the BLM was produced using the painting method [23,24]. The electrical circuit was completed with two Ag/AgCl electrodes inserted in the electrolyte solutions and wired to the headstage of an Axopatch200B electrophysiology amplifier (Molecular Devices, San Jose, CA, USA). The analog signal was fed into a DigiData 1440A digitizer (Molecular Devices) connected to a personal computer for signal monitoring and data recording. The BLM formation was monitored from the membrane capacitance inferred from the amplitude of the rectangular capacitive current measured (at 4 μs sampling rate) in response to a triangle-shaped stimulatory voltage provided by a function generator. For this experimental system, a capacitance in the range 60–80 pF provided long-term stability; lower capacitances led to formation of multilayers, while higher capacitances led to premature membrane rupture upon exposure to a differential hydrostatic pressure. The membrane conductance was determined from the slope of the IV plots recorded in response to voltage ramps ranging from −20 mV to +20 mV at a voltage change rate of 2 mV/s and a sampling rate of 0.1 s. The constant capacitive component (I_c_ = CdV/dt) was subtracted from the recorded currents to eliminate the y offset of the IV plots (which does not affect their slopes).

**Figure 2:**
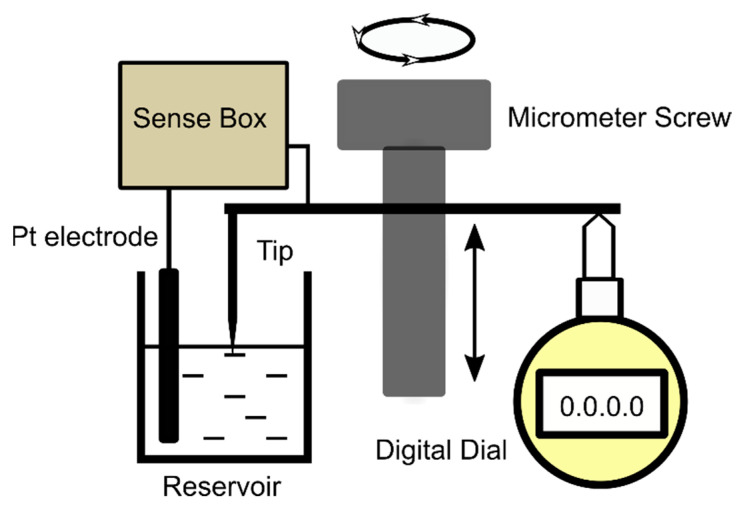
The simplified diagram (not to scale) of experimental setup utilized for constructing the plot height vs. added volume and calibration.

The procedure for achieving pressurized membranes (Figure 1b) was initiated by creating a differential pressure *p* = ρg∆h on the two sides of the membrane, where ρ is the electrolyte density (1.007 ± 0.0015 g/mL, determined by weighing 1 mL of solution, *n* = 5), g is the gravitational acceleration, and ∆h is the height difference between the electrolyte solution levels in the two reservoirs. Upon increasing the volume of the solution in the grounded reservoir, we assumed that the additional pressure leads to a curved membrane, with a different geometry from a planar one. This difference in geometry can be exploited for qualitative description of changes in surface area, as described in the results section. For the purpose of quantitative evaluations on the differential pressure, we used a custom-made system to measure the height of the electrolyte upon successive additions and subtractions of fluid into and from reservoirs (for this experiment, a PTFE partition with no hole was used between the assembled Delrin^®^ reservoirs). The core of the setup depicted in Figure 2 is a micrometer screw that moves a plate in contact with the probing rod of a digital dial indicator (1 μm resolution, DITR-0055 Digital Indicator, Clockwise Tools) at the same time with a very sharp tip (the curvature of the tip was less than 10 μm) made of stainless steel and positioned in the center of the reservoir to avoid the menisci formed at the solution–wall interfaces. A custom sense box containing a current amplifier and audio/visual indicators was used to precisely detect when the sharp tip made contact with the electrolyte solution; for completing the electrical circuit, another Pt wire served as an electrode continually embedded into the bulk solution. This device was utilized to calibrate each reservoir (from the plot of height vs. added volume) for both insertion and retraction of the tip into and from solution. For additions, the reservoirs were prefilled with 200 μL electrolyte solution to ensure proper electrical connections and a flat solution-air interface.

Electrophysiology data recording and preliminary analysis (capacitance and conductance) were performed with the pClamp10.0 software package (Molecular Devices); the data were further analyzed and plotted with Origin 8.5.1 (OriginLab Corporation, Northampton, MA, USA). All the experiments were performed at room temperature (22 ± 1 °C).

## 3. Results and Discussion

### 3.1. Height Calibration of the BLM Reservoir

**Figure 3:**
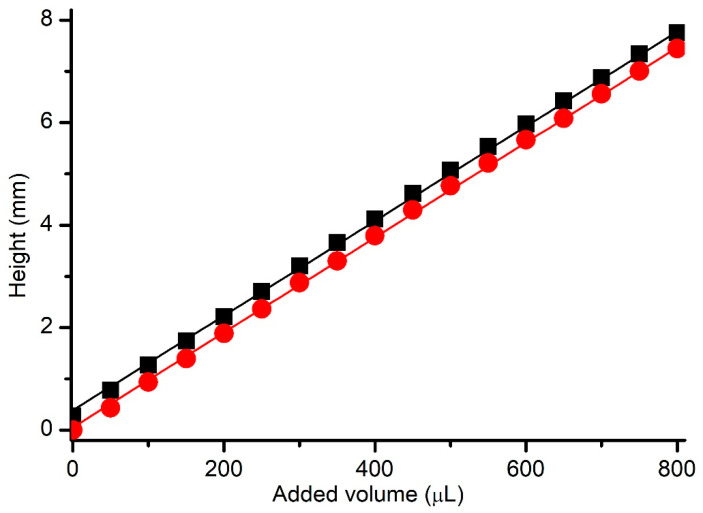
Determination of the relationship between the height of the solution column and added volume. The height measurements were performed by inserting the tip into solutions (red symbols) or retracting it (black symbols). The straight lines represent the linear fit of the experimental data for the two situations. The symbols represent averaged points from triplicate experiments performed on four reservoirs; the standard deviations are smaller than the size of the symbols.

The first investigations aimed at calibrating the chamber with respect to the hydrostatic pressure and determining the replicability for multiple electrolyte additions and removals. Both pairs (a total of four identical reservoirs) were tested to ensure that interchangeability between chambers did not influence the measurements. The custom experimental setup was utilized to determine the height of the electrolyte column, as described in the Methods section. Addition of the electrolyte to the reservoirs led to an increase in height, which was measured with the digital indicator. When the first set of additions was finalized, we also measured the height upon removal of the solution from the reservoir. The experiments were carried out in triplicate for all four reservoirs. The plot showing height as a function of added volume (Figure 3) indicated an excellent linearity for all reservoirs, as well as a nearly identical slope of 9.2 μm/μL. The differences between the y-intercepts observed for addition and removal can be easily explained by considering the meniscus that formed when the sharp probing tip was removed from the solution, which led to the formation of a conducting channel between the tip and solution’s surface [25].

### 3.2. Preliminary Investigations on the Influence of Differential Pressure on Membranes’ Conductance

**Figure 4:**
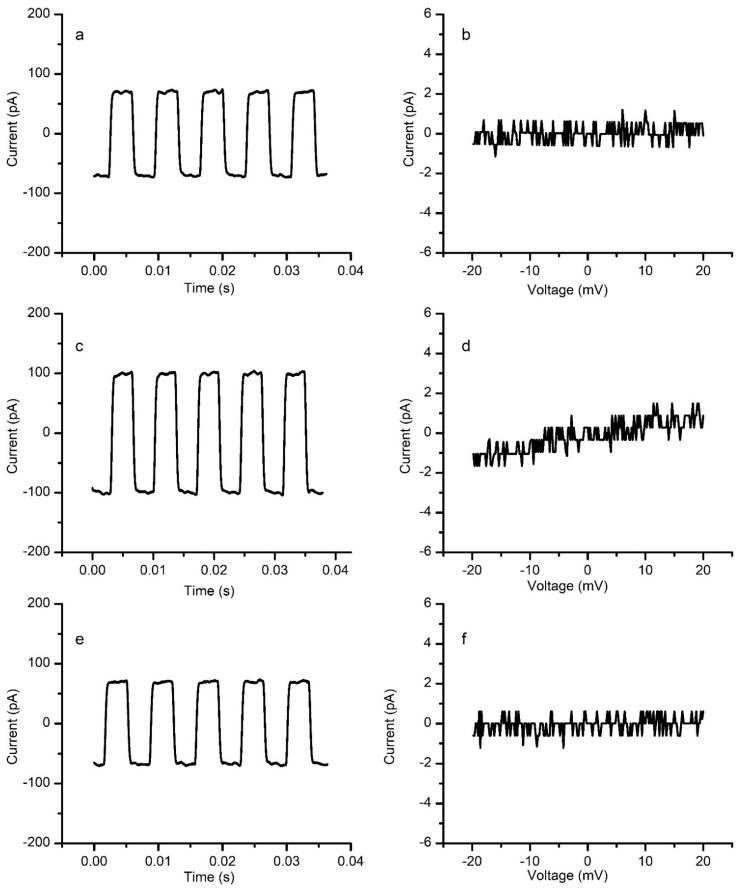
Preliminary testing of the experimental system on a cholesterol-free membrane. A relaxed membrane has a capacitance of ~70 pF (**a**) and a very low conductance (**b**). Pressure from the additional solution in the grounded reservoir leads to an increase in capacitance (**c**) and conductance (the slope of the IV plot) (**d**). The elimination of the differential pressure by solution removal restores the initial capacitance (**e**) and conductance (**f**).

Next, we proceeded with preliminary setup testing by observing the influence of the differential hydrostatic pressure on the conductance of a cholesterol-free membrane. The membrane capacitance measured from the amplitude of the capacitive current in the absence of pressure (no electrolyte additions) was ~70 pF (Figure 4a), and the conductance inferred from the IV plot (Figure 4b) was under 0.01 nS (corresponding to a 100 GΩ membrane, indicative of an excellent seal). A crude estimation of the specific capacitance and conductance by considering a membrane diameter of ~110 µm provides estimated values well within the range reported in the literature, for both specific capacitance and conductance (i.e., capacitance over 0.6 μF/cm^2^ and conductance of ~110 nS/cm^2^ [26,27]). Upon addition of 100 μL electrolyte solution to the grounded reservoir, the membrane’s capacitance suddenly increased to ~100 pF (Figure 4c) and the membrane’s conductance increased and attained a value of ~0.06 nS (as inferred from the IV plot depicted in Figure 4d). Removal of 100 μL solution from the same reservoir in which the addition was performed led to recovery of both capacitance (~70 pF, Figure 4e) and conductance (~0.01 nS, Figure 4f).

A reasonable explanation for the changes in conductance may be provided by considering the effects of applied pressure. The addition of electrolyte solution to the grounded reservoir led to the development of a differential pressure that pushed the membrane from one side. This push led to an increased capacitance, which indicates a significant change in membrane geometry. The changes in the membrane’s capacitance may originate in a larger surface area, bilayer thinning, or both. Previously reported simulations indicate that a curved membrane may undergo thinning [19]; since this is predicted to be very small [19], we concluded that the change in the surface area of a pressurized membrane is the major contributor to the increased capacitance. However, we may not disregard the serious gap in our knowledge regarding the absence of conclusive information with respect to how the planar lipid membranes increase their surface area in response to pressure. A bilayer lipid membrane is an open system, in which the Plateau-Gibbs (PG) border (annulus) [28,29] plays the role of a lipid reservoir, an essential feature for the membrane’s stability. However, we do not know if an increased surface area of the curved membrane is attained by employing lipids from the PG border, or if the border behaves like a fixed edge and the only effect is the curving of the membrane. While it seems reasonable to assume that the bilayer membrane undergoes curving upon application of differential hydrostatic pressures, irrespective of changes at the PG border, such changes may lead to additional variations in capacitance and conductance. Imaging may provide information on the size of the membrane and annulus, but this would be accurate only for the flat membrane. Bright-field microscopy may provide only a 2D projection of a 3D structure, impeding a correct determination of the changes in geometry. While curvatures may be easily visualized at larger scales [26], precise assessments at microscale may be accomplished by employing Confocal Laser Scanning Microscopy [30], thus, enabling the modelling of individual contributions from the membrane and annulus. Another issue may be represented by the short-chain solvent (i.e., n-decane) used to solubilize the lipids, which may remain embedded in the self-assembled structure, modulating its mechanical and electrical properties. In this respect, substantial membrane thinning (leading to increased capacitance) may originate in the solvent exclusion from membranes curved by pressure, but this may not be reversible. Despite all these shortcomings, we hypothesized that the major contributor to the observed changes in capacitance of a membrane exposed to small differential hydrostatic pressure was the change in surface area due to pressure-induced curving. This hypothesis, though it needs further verification, is supported by the observation that the recovery of the original electrical properties (i.e., capacitance and conductance) was very fast for reasonable values in the capacitance of membranes exposed to pressure. For this lipid composition, capacitances up to ~300 pF attained upon solution additions enabled a fast recovery after removal of the additional solution volumes, while over this capacitance value, the recovery was much slower (up to tens of minutes) or completely abrogated (the initial capacitance was not reinstated even after tens of minutes). We understand that the fast relaxation of a stressed membrane does not exclude a rapid exchange of lipids with the PG border; nonetheless, the membranes exposed to differential hydrostatic pressure very likely still present curvature and additional intramembrane mechanical stress, validating our experimental results for the conditions described in this work.

### 3.3. Pressure Influences the Ionic Conductance of Cholesterol-Free Membranes

Next, we investigated the relationship between the cholesterol-free membrane capacitance and conductance for an entire range of applied pressures. With all our efforts, it was impossible to conduct independent experiments in which the initial conditions (i.e., conductance and capacitance) were identical. This is not necessarily a consequence of the different geometrical parameters of the hole produced in the PTFE film (these were verified by microscopy, and were consistent); therefore, we assumed that the differences in initial capacitances originated in different sizes of the PG border. In the same line, much smaller differences (yet not negligible) were measured for the initial conductance of relaxed membranes. Therefore, to facilitate data comparison between independent experiments, we opted for plotting the variation in these parameters (both against initial values measured for the relaxed membranes) for each of the experimental conditions; whatever the case, membranes with an initial conductance much larger than 0.01 nS (i.e., 100 GΩ) were discarded to avoid artifacts arising from substantial leakage.

**Figure 5:**
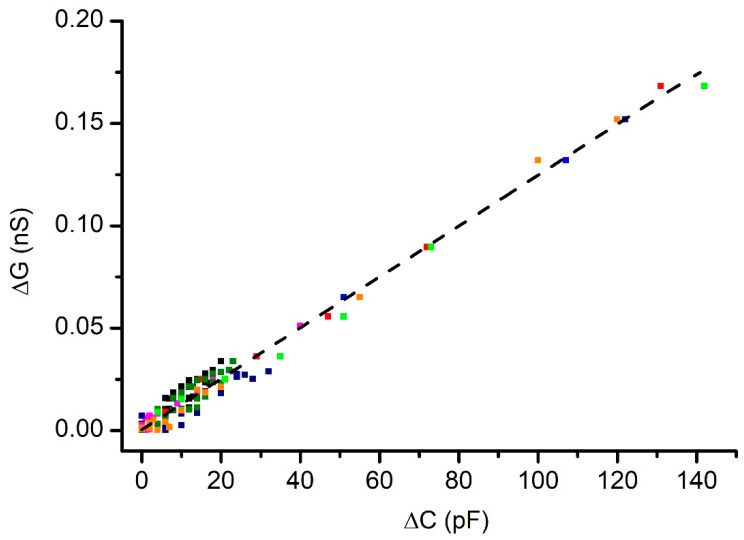
The changes in conductance correlate linearly with the changes in capacitance for a cholesterol-free membrane under differential hydrostatic pressure. The interrupted line represents the linear fit of the experimental data; the different colors used for symbols indicate independent experiments.

The results for cholesterol-free membranes (plotted in Figure 5) show a quasi-linear relationship between the changes in conductance and capacitance. Each point in the plot indicates the average value, determined from at least two experiments carried out on the same membrane; however, the plot includes the results from eight membranes, in which we encountered slight variations in initial electrical parameters. In addition, the experimental values were determined after additions and subtractions of electrolyte solutions from the grounded reservoirs for each membrane, suggesting good reversibility.

An excellent linear correlation between changes in capacitance and conductance was determined for all the data plotted in Figure 5; this was indicated by a Pearson correlation factor of 0.99 and an R^2^ value of 0.98. Quite interesting is the observation that very small changes in the differential pressure led to unexpectedly large adjustments in the capacitance. The slope of the plot presented in Figure 3 enables the calculation of the differential hydrostatic pressure after each change in height. For the reservoirs used, the addition of 25 µL electrolyte led to measurable changes in capacitance and conductance, although the hydrostatic pressure difference was only ~230 µm solution column, corresponding to ~2.27 Pa (those values were estimated based on the plot depicted in Figure 2, and the estimated density of the solution). The similar changes in the measured electrical parameters of curved membranes, for which the starting PG border was different, suggests that the small, applied pressure most probably curved the membranes without excessive use of lipids from the PG reservoir.

### 3.4. Effects of Pressure on the Conductance of Membranes with Cholesterol

**Figure 6:**
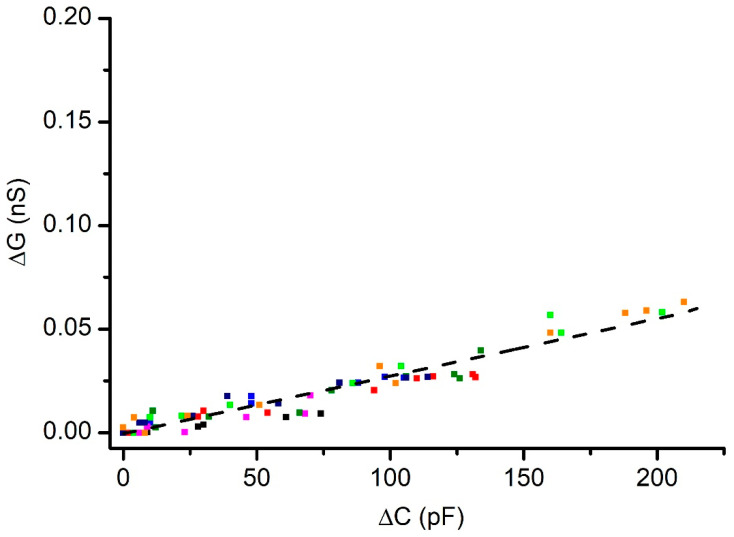
A membrane containing cholesterol undergoing changes in conductance and capacitance upon exposure to differential hydrostatic pressure. Cholesterol addition leads to diminished changes in conductance even for substantial variations in capacitance. The interrupted line represents the linear fit of the experimental data, and the different colors used for symbols indicate independent experiments.

The experiments carried out on membranes containing cholesterol led to qualitatively similar results, but the quantitative parameters were substantially different (Figure 6). A quasi-linear relationship between changes in conductance and capacitance was also observed but the changes in conductance were much smaller than what we determined for a cholesterol-free membrane undergoing similar changes in capacitance (as shown in Figure 5). This result indicates that pressure-curved membranes containing cholesterol are less permeable to inorganic ions, which confirms the well-established effects of cholesterol in adjusting the membrane’s transport properties [31,32]. Similar to the experiments that employed cholesterol-free membranes, a satisfactory linear match between changes in conductance and capacitance was also observed for the membranes with cholesterol, as indicated by a Pearson’s correlation factor of 0.96, and an R^2^ value of 0.93.

**Figure 7:**
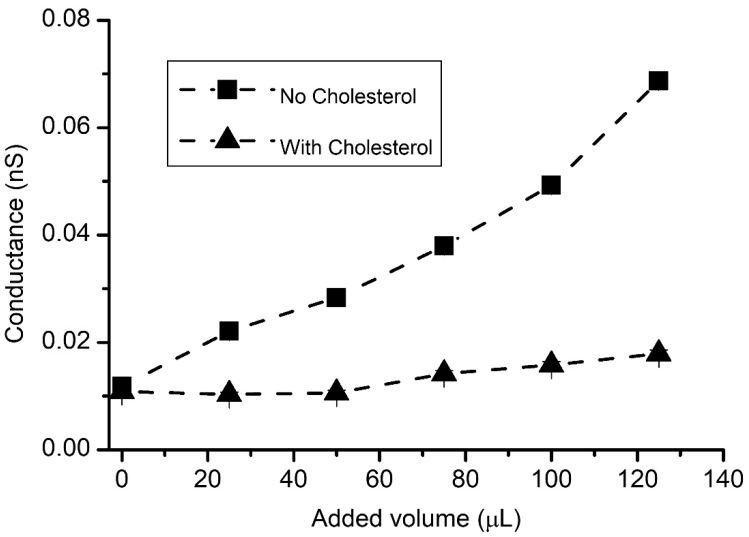
Cholesterol modulates the changes in membrane’s conductance in response to hydrostatic pressure created by solution additions. The conductance varies differently for membranes without cholesterol (squares) and with cholesterol (up triangles). The average conductance values were calculated from three different runs of the same experiments.

Besides the reduced permeability, another important feature was inferred from our experimental observations. The changes in conductance and capacitance for a cholesterol-free membrane started to manifest after only small adjustments in the solution volume, typically ~25 μL, while the membranes with cholesterol required additions of ~100 μL for initiating similar changes. In addition, the cholesterol-free membrane underwent frequent ruptures upon addition of a few hundred µL of solution, while the membrane with cholesterol displayed an enhanced stability for additions approaching 1 mL. The repeatability of the changes in capacitance and/or conductance upon identical solution additions was rather poor between independent experiments; the results from single experiments presented in Figure 7 show that small volume solution additions had a greater effect on the conductance of the cholesterol-free membrane compared to membranes containing cholesterol. Quite often, small additions led to small, yet significant, changes in capacitance, not accompanied by significant changes in conductance, especially for the membranes with cholesterol (which is also seen in Figure 5 and Figure 6). Nonetheless, once the differential pressure increased, the variations in capacitance and conductance varied in a quasi-linear manner for both lipid compositions. This confirmed the well-known role played by cholesterol in establishing the membrane’s fluidity and elasticity [33,34,35,36,37,38], which are important mechanical parameters, contributing to the overall membrane’s stability.

Our investigations point out a relationship between the geometrical changes induced on a planar lipid membrane by application of a controlled differential pressure and its transport properties. Although our experiments employed membranes with solvent, which may adjust electrical and mechanical properties, it may be easily utilized for solvent-free membranes, thus, enabling understanding regarding the effects of various solvents on the ionic permeability of curved membranes. We consider that major limitations of the approach proposed in our work arise from equating the changes in conductance to changes in capacitance, as opposed to changes in mechanical stress. For an ideal system, a relationship between the capacitance and curvature of a spherical cap may be easily modeled; unfortunately, a simple estimation of the stress in the membrane from capacitance measurements is not an easy task. It is not clear if the enlarged surface employs pulling lipids from the PG border, so a fixed edge model may not be realistic. In the same line, the changes in thickness upon curving are not known; although they have been estimated to be less than 1 nm [19], they may be much larger for a membrane with n-decane as the solvent and contribute significantly to the observed changes in capacitance. The asymmetrical distribution of membrane components, either between leaflets [19] or owing to organization into lipid rafts [39,40], may introduce additional limitations for adopting one of the largely utilized membrane bending models [41,42,43]. A more feasible approach would be the utilization of membrane tension indicators [44,45,46,47,48,49], in combination with an easier-to-model artificial system (i.e., giant vesicles exposed to osmotic pressure [45]) for calibration and direct measurements on curved membranes. The experiments presented in this work do not provide direct clues for identifying a particular permeation mechanism from the several that are available [15,16]; nonetheless, a comparison between predictions from simulations and experimental results may help with such identification.

In conclusion, despite its shortcomings, the proposed experimental setup enabled investigation into the membrane’s permeability to inorganic ions, upon exposure to mechanical stress induced by the differential pressure. This may prove useful for a better understanding of how mechanical factors modulate the permeability of lipid membranes, as well as the transport of ions and molecules, through specialized transporters reconstituted into artificial membrane systems. This is anticipated to provide insight into how mechanics contribute to the modulation of a large variety of biological activities and cellular functionalities, adjusted by transmembrane transport phenomena.

## Data Availability

The raw datasets recorded and analyzed during this study are available from the corresponding author on reasonable request.
